# Multifunctional BiOI/SiO_2_/Fe_3_O_4_@*n*-Docosane Phase-Change Microcapsules for Waste Heat Recovery and Wastewater Treatment

**DOI:** 10.3390/ma16041656

**Published:** 2023-02-16

**Authors:** Jianwei Jing, Huan Liu, Xiaodong Wang

**Affiliations:** State Key Laboratory of Organic–Inorganic Composites, Beijing University of Chemical Technology, Beijing 100029, China

**Keywords:** phase-change materials, magnetic microcapsules, BiOI nanosheets, waste heat recovery, wastewater treatment

## Abstract

Waste heat and organic contaminants are significant issues in water pollution, which has caused ecological problems and threatened human health. To provide an effective solution for wastewater recovery, we designed a novel type of multifunctional phase-change microcapsule. This type of microcapsule was synthesized using *n*-docosane as a core and a SiO_2_/Fe_3_O_4_ composite as a base shell through in situ interfacial polycondensation with the assistance of a Fe_3_O_4_ nanoparticle as a Pickering emulsion stabilizer, followed by the deposition of BiOI nanosheets on the surface of the SiO_2_/Fe_3_O_4_ composite shell. Benefiting from the *n*-docosane core, the resultant microcapsules obtained phase-change enthalpies of 46.8–115.7 J/g for absorbing waste heat from wastewater. The deposited BiOI nanosheets promoted photocatalysis for the microcapsules to degrade organic contaminants in wastewater. Owing to the magnetic response of the Fe_3_O_4_ nanoparticles, the separability and recyclability of the microcapsules were improved significantly by magnetic separation. Moreover, the microcapsules demonstrate outstanding phase-change reversibility, thermal cycling stability, and shape stability due to the tight SiO_2_/Fe_3_O_4_ composite shell. This study provides a promising approach for designing and developing multifunctional phase-change microcapsules for waste heat recovery and wastewater treatment.

## 1. Introduction

Solid–liquid phase-change materials (SLPCMs) are a type of heat energy storage material that can store and release a large amount of heat energy through reversible, solid-to-liquid phase transitions [1]. SLPCMs have distinguishing features, including a high energy storage capacity, nonreactivity, and almost constant temperature during phase transitions [2,3]. These distinguishing characteristics endow SLPCMs with applications in solar energy storage and utilization, building energy conservation, waste heat recovery, pharmaceutical product preservation, air-conditioning systems, and the thermal management of Li-ion batteries [4,5]. Generally, SLPCMs are categorized as organic or inorganic SLPCMs. Inorganic SLPCMs have the advantage of an outstanding latent heat, low prices, and stable security. However, some inherent disadvantages of inorganic SLPCMs, such as supercooling and phase separation, limit their large-scale applications. These advantages lead to a poor recycling stability and a delay in the thermal response of inorganic SLPCMs for heat energy storage and release [6]. Compared to inorganic SLPCMs, organic SLPCMs exhibit a lower supercooling degree and better phase stability. Therefore, organic SLPCMs such as paraffin waxes (PWs) are considered the most attractive candidate for application in heat energy storage and temperature management. Nevertheless, as typical organic SLPCMs, PWs are exposed to problems with leakage and low thermal conductivity [7,8].

To address the leakage behavior, scientists have proposed a microencapsulation technology for packaging SLPCMs in a micro-container to achieve good shape stabilization [9,10]. Shell materials provide the encapsulated SLPCMs with good shape stability, high thermal cycle stability, a large heat transfer area, and a relatively constant volume, which can effectively prevent the leakage of SLPCMs. Based on the chemical properties of the shell materials, they can be divided into organic materials (melamine formaldehyde resin, urea formaldehyde resin, polyurea, acrylic, polyaniline, and organo-silica resins), organic–inorganic hybrid materials (polymethyl methacrylate/SiO_2_, polystyrene/SiO_2_, urea formaldehyde resin/TiO_2_, and melamine formaldehyde resin/graphene), and inorganic materials (SiO_2_, TiO_2_, CaCO_3_, Al_2_O_3_, graphene, and carbon nanotubes) [11]. The organic-based SLPCM microcapsules have certain limited applications due to the flammable features and low thermal conductivity of organic-based shells [12]. In comparison to organic-based shells, inorganic-based shells demonstrate nonflammability, better thermal conductivity, higher mechanical strength, and higher stiffness [13,14]. Therefore, the inorganic-based SLPCM microcapsules exhibited a fast thermal response and an enhanced heat transfer for reversible and recyclable heat energy storage and release, which have been widely studied in recent years. Moreover, the smart combination of SLPCMs with functional metal oxides (VO_2_, PbWO_4_, TiO_2_, SnO_2_, and Fe_3_O_4_) can not only impart a good shape stability and high thermal stability to the resultant SLPCM microcapsules but also imparts them with a variety of extra functions, including electrochemical energy storage, gamma radiation shielding, UV shielding, photocatalysis, antibiosis, and magnetic response [15,16]. These functional SLPCM microcapsules present great potential in developing an advanced renewable energy infrastructure. In our previous study, a type of Fe_3_O_4_/CaCO_3_-based, PW microcapsule system was designed for wastewater recovery [17,18]. With its high latent heat capacity, the encapsulated PW can absorb and store a large amount of waste heat. Synchronously, the CaCO_3_ shell not only acts as a compact shell for the prevention of encapsulated PW leakage but also enables the microcapsules to implement the removal of organic dyes and heavy metal ions from wastewater. Moreover, the Fe_3_O_4_ nanoparticles in the CaCO_3_ shell provide an excellent magnetic response for the microcapsules to promote their reusability and recyclability. However, the CaCO_3_ shell has limited adsorption capacities for adsorbing heavy metal ions and organic dyes. As a result, the microcapsules need to undergo a washing process for continuous wastewater recovery. The washing process complicates the practical application of the Fe_3_O_4_/CaCO_3_-based, PW microcapsules and may result in secondary pollution. On the other hand, the CaCO_3_ shell exhibits poor acid resistance, making it unsuitable for wastewater treatment under acidic conditions.

In this study, we designed a type of BiOI/SiO_2_/Fe_3_O_4_@*n*-docosane, phase-change microcapsule to provide an effective solution for wastewater recovery under various harsh conditions. The phase-change microcapsules were fabricated by microencapsulating *n*-docosane as an SLPCM core in a SiO_2_/Fe_3_O_4_ composite shell, followed by the surface deposition of BiOI nanosheets. With its high energy storage capacity, the *n*-docosane core can store a large amount of waste heat from wastewater. The SiO_2_/Fe_3_O_4_ composite shell provides adequate protection for the *n*-docosane core, preventing its leakage during the solid–liquid phase transition and endows the phase-change microcapsules a high thermal conductivity, enhancing their thermal response. The Fe_3_O_4_ nanoparticles on the composite shell contribute a magnetic response for the microcapsules and facilitate their efficient separation for wastewater treatment. As a p-type semiconductor, BiOI has a specific, layered crystal structure, a narrow band gap (1.73–1.92 eV), and good chemical stability. The conduction of photogenerated carriers can be accelerated by its unique (Bi_2_O_2_)^2+^ layer structure [19]. More importantly, BiOI demonstrates a strong absorption of visible light; this is superior to TiO_2_ photocatalysts such as the commercial product P25, which has a band gap of 3.2 eV [20]. The wide band gap leads to a restriction of excitation of TiO_2_ under UV light irradiation and decreases its utilization of sunlight. The superior photocatalysis of BiOI in visible light makes it a promising photocatalyst for the removal of organic contaminants in wastewater such as methylene blue [21], Rhodamine B [22], phenol [23], tetracycline [24,25], and bisphenol A [26]. Nevertheless, BiOI with a 2D, lamellar structure is liable to agglomerate, leading to restricted active sites. The restricted active sites decrease the adsorption capacity of BiOI for a target pollutant [27]. Moreover, BiOI exhibits the disadvantages of poor electric conductivity, low photo-oxidation ability, and a high recombining tendency of photo-generated electron–hole pairs, limiting its practical applications. Numerous effects have been conducted to promote the photocatalytic activity of BiOI by combining with TiO_2_ [28], Ag [29], CdS [30], BiPO_4_ [31], WO_3_ [32], and carbon quantum dots [33]. Given these prominent characteristics, the phase-change microcapsules developed in this study obtained phase-change enthalpies of 46.8–115.7 J/g for absorbing waste heat from wastewater and photocatalytic activity for organic contaminant removal, including methyl orange, Congo red, and tetracycline. Moreover, the phase-change microcapsules exhibited outstanding phase-change reversibility, thermal cycling stability, and shape stability. This study offers a promising approach for highly efficient waste heat recovery and wastewater treatment.

## 2. Materials and Methods

### 2.1. Materials

*N*-docosane, used as a paraffin-type SLPCM, was obtained from Shanghai Macklin Biochemical Co., Ltd., Shanghai, China. Tetraethoxysilane (TEOS, 98 wt%), ferric chloride hexahydrate (FeCl_3_·6H_2_O, 99.0 wt%), cetyltrimethyl ammonium bromide (CTAB, 98 wt%), ferrous chloride tetrahydrate (FeCl_2_·4H_2_O, 99.7 wt%), ammonia (NH_3_·H_2_O, 25.0–28.0 wt%), HCl aqueous solution (37.5 wt%), and formamide (99 wt%) were commercially provided by Beijing Chemical Reagents Company, Beijing, China. Congo red (99 wt%), methyl orange (99 wt%), tetracycline (99 wt%), bismuth nitrate pentahydrate (Bi(NO_3_)_3_·5H_2_O, 99 wt%), and potassium iodide (KI, 99 wt%) were commercially supplied by Tianjin Fuchen Chemical Reagent Co., Ltd., Tianjin, China. All chemicals were used as received without further purification.

### 2.2. Synthesis of Phase-Change Microcapsules

A co-precipitation of Fe^2+^- and Fe^3+^-synthesized Fe_3_O_4_ nanoparticles was produced according to our previous study [17]. In a typical process, a 250 mL, three-necked flask was charged with 1.08 g of FeCl_3_·6H_2_O, 0.46 g of FeCl_2_·4H_2_O, and 100 mL of deionized water. It was then heated to 55 °C with stirring for 25 min to achieve an aqueous solution. Next, 4.0 mL of NH_3_·H_2_O was added to the flask with continuous agitation for 20 min. With the co-precipitation completed, the Fe_3_O_4_ nanoparticles were collected by a magnet, washed with water several times, and dispersed in 40 mL of formamide to obtain an Fe_3_O_4_ suspension.

**Figure 1:**
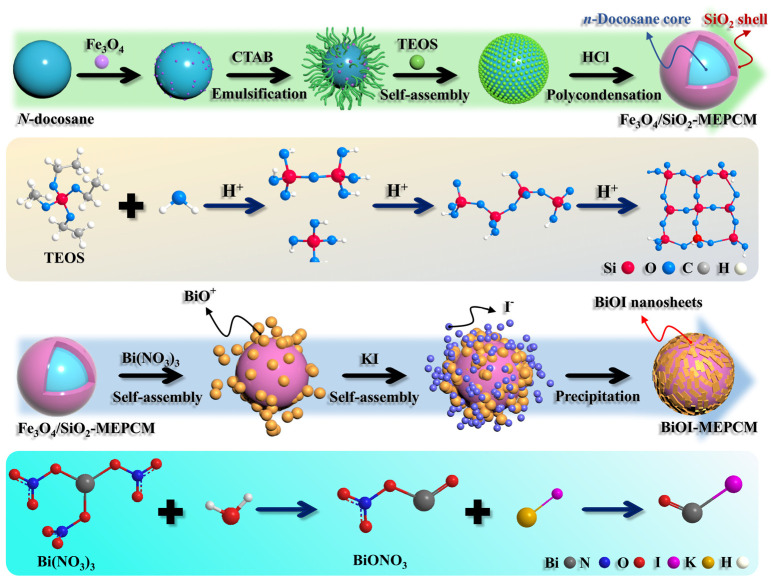
Scheme of synthetic procedure and reaction mechanism of BiOI-MEPCM.

The phase-change microcapsules with a SiO_2_/Fe_3_O_4_ composite shell and an *n*-docosane core (hereafter named SiO_2_/Fe_3_O_4_-MEPCM) were fabricated as base microcapsules according to the synthetic strategy shown in Figure 1. In a typical procedure, a 500 mL, three-neck flask was charged with 10.0 mL of Fe_3_O_4_ suspension, 4.0 g of *n*-docosane, and 4.0 g of TEOS with agitation at 55 °C for 1.5 h. Next, 0.8 g of CTAB was dissolved in 100 mL of formamide at 55 °C and added to the flask with continuous stirring for 2.5 h to obtain a stable, oil-in-water emulsion. Then, 60.0 mL of HCl solution (pH: −0.24) was added dropwise into the flask to initiate the hydrolysis of TEOS and the interfacial polycondensation of hydrated silica with agitation at 55 °C for 4 h. This was then aged for 12 h. With the reaction completed, the SiO_2_/Fe_3_O_4_-MEPCM was collected by a magnet, washed with water several times, and dried at room temperature for 12 h.

The SiO_2_/Fe_3_O_4_-MEPCM, deposited with BiOI nanosheets (hereafter named BiOI-MEPCM), was synthesized based on the synthetic strategy presented in Figure 1. In a typical procedure, 0.5 g of SiO_2_/Fe_3_O_4_-MEPCM, 1.0 mmol of Bi(NO_3_)_3_·5H_2_O, and 120 mL of deionized water were added to a 250 mL, three-necked flask with stirring at 80 °C for 30 min. Next, 1.0 mmol of KI, 0.4 g of CTAB, and 60 mL of deionized water were added to a beaker, ultrasonicated for 10 min, and then added dropwise to the flask with agitation for 30 min. Subsequently, NH_3_·H_2_O was added to the flask with continuous agitation for 3 h to maintain a pH of approximately 7 for the mixture solution. The BiOI-MEPCM was collected by a magnet, washed with water three times, and dried at room temperature for 12 h. During the synthetic production of BiOI-MEPCM, the mass ratios of the SiO_2_/Fe_3_O_4_-MEPCM and the BiOI nanosheets were set as 1.5:1, 1:1, and 0.5:1 and were named BiOI-MEPCM (1.5:1), BiOI-MEPCM (1:1), and BiOI-MEPCM (0.5:1), respectively. In addition, BiOI-MEPCM without an *n*-docosane core synthesized under the identical condition and named BiOI-sphere for comparison.

### 2.3. Characterizations

The morphologies of SiO_2_/Fe_3_O_4_-MEPCM and BiOI-MEPCM were characterized by using a field emission scanning electron microscope (SEM, TM3030, Hitachi, Tokyo, Japan). The *X*-ray photoelectron spectroscopy (XPS) spectra of BiOI-MEPCM were determined by an *X*-ray photoelectron spectrometer (ESCALAB 250Xi, Thermo Scientific, Waltham, MA, USA). The *X*-ray diffraction (XRD) patterns were characterized by an *X*-ray diffractometer (D/max 2500, Rigaku, Tokyo, Japan) under Cu Kα radiation. The Fourier-transform infrared (FTIR) spectra of pure *n*-docosane, SiO_2_/Fe_3_O_4_-MEPCM, and BiOI-MEPCM were evaluated by an infrared spectrometer (Nicolet iS5, Thermo Scientific, USA) with a resolution of 2 cm^−1^. The specific surface area was determined by a sorptometer (ASAP 2020, Micromeritics, Norcross, GA, USA) at 77 K using the Brunauer–Emmett–Teller (BET) model. The magnetic response of the BiOI-MEPCM was evaluated using a vibrating sample magnetometer (VSM-7410, Lake Shore, Westerville, OH, USA) at an applied field range from −20,000 to 20,000 Oe.

A differential scanning calorimeter (DSC, Q20, TA Instruments, New Castle, DE, USA) determined the thermophysical property and thermal reliability at a heating/cooling rate of 10 °C/min with a N_2_ flow of 60 mL/min. The thermal stability was evaluated using a thermogravimetric analyzer (TGA, Q50, TA Instruments, USA) at a heating rate of 10 °C/min under a N_2_ atmosphere with a gas flow of 50 mL/min. The heat energy storage performance of the BiOI-MECPM was characterized by an infrared thermographic camera (875–1i, Testo™, Lenzkirch, Germany). The shape stability was characterized by isothermal heating analysis. Pure *n*-docosane and the phase-change microcapsules were placed on a high-precision electronic heating platform. A digital camera was used to record the shape of the samples. Before the heat energy storage and shape stability characterizations, pure *n*-docosane and phase-change microcapsule powders were pressed by a pressure machine with an intensity of 5.0 MPa for 3 min to obtain test samples with a diameter of 1.35 cm. The photocatalytic activity of the BiOI-MEPCM was determined by photodegrading methyl orange, Congo red, and tetracycline as three representative organic contaminants under an ambient temperature of 17–22 °C and humidity of 25–50%. In a typical procedure, 1000.0 mL of deionized water (pH: 6.8) and 50.0 mg of organic contaminant were mixed to obtain an organic contaminant solution (*C*_0_, 50 mg/L). Next, 100 mL of organic contaminant solution and 10 mg of the phase-change microcapsules were added to a beaker and stirred using a magnetic stirrer in a dark environment for 120 min to achieve an adsorption–desorption equilibrium. Afterward, a Xenon lamp, used as a solar simulator, served as a light source for illuminating the beaker. The concentration of organic contaminant solution (C, mg/L) was analyzed by a UV–visible spectrophotometer (UV-2550, Shimadzu, Kyoto, Japan) at given intervals of illumination time.

## 3. Results and Discussion

### 3.1. Synthetic Strategy and Morphology

**Figure 2:**
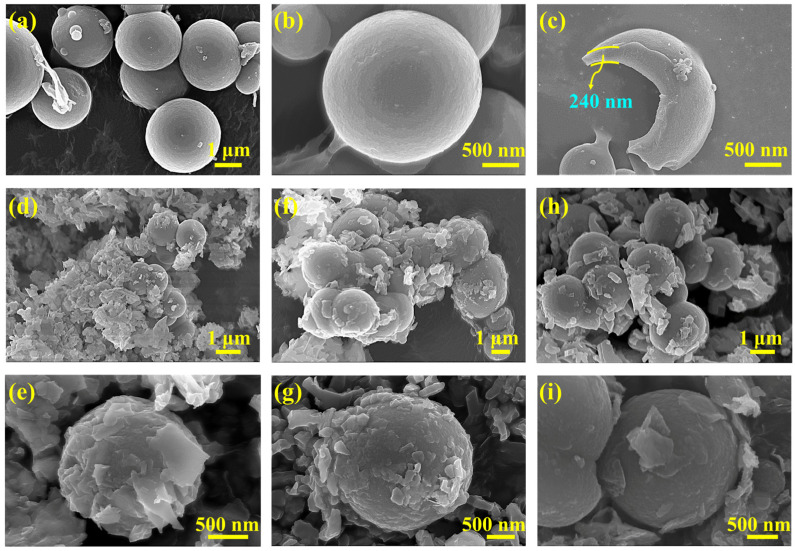
SEM micrographs of (**a**–**c**) SiO_2_/Fe_3_O_4_-MEPCM, (**d**,**e**) BiOI-MEPCM (1.5:1), (**f**,**g**) BiOI-MEPCM (1:1), and (**h**,**i**) BiOI-MEPCM (0.5:1).

Figure 1 shows the synthetic procedure and mechanism of BiOI-MEPCM. The BiOI-MEPCM comprised an internal *n*-docosane core, an intermediate protective and supportive, inorganic SiO_2_/Fe_3_O_4_ composite shell, and an external photocatalytic layer of BiOI nanosheets created using a two-step encapsulation process, including the in situ interfacial polycondensation of TEOS and the precipitation reaction of BiOI. Fe_3_O_4_ nanoparticles were selected as a Pickering stabilizer to obtain a stable *n*-docosane emulsion system and as a magnetic response material to provide magnetic separation for the BiOI-MEPCM. SiO_2_ was used as a compact shell to encapsulate the *n*-docosane core due to its sufficient mechanical strength and high thermal conductivity. According to our previous study, the *n*-docosane core and TEOS can be set at a mass ratio of 1:1 to achieve a high heat storage capacity and physically stable structure for the SiO_2_/Fe_3_O_4_-MEPCM [34]. As is shown in Figure 2a,b, the SiO_2_/Fe_3_O_4_-MEPCM exhibited a spherical morphology with a smooth surface. After undergoing pressing in a pressure machine with an intensity of 10.0 MPa and manual grinding, the SiO_2_/Fe_3_O_4_-MEPCM showed a perfect core–shell structure. Using a SEM micrograph of a cracked microcapsule (Figure 2c), its shell thickness was determined to be 240 nm. With the successful synthesis of SiO_2_/Fe_3_O_4_-MEPCM, BiOI nanosheets were prepared for deposition onto the surface of the SiO_2_/Fe_3_O_4_-MEPCM. Firstly, BiO^+^ was self-assembled onto the surface of the SiO_2_/Fe_3_O_4_-MEPCM through the electrostatic attraction between BiO^+^ and the negative charge of SiO_2_. I^−^ was then accumulated on the microcapsule’s surface. By controlling the pH of the reaction mixture, a precipitation reaction between BiO^+^ and I^−^ took place to form BiOI nanosheets. With the deposition of BiOI nanosheets, the microcapsules exhibited a considerably rough surface (Figure 2d,f,h). Moreover, the roughness of the BiOI-MEPCM surface was increased with the increase in BiO^+^ and I^−^ (Figure 2e,g,i). Understandably, an increase in BiO^+^ and I^−^ led to more BiOI nanosheets being deposited onto the SiO_2_/Fe_3_O_4_-MEPCM surface. As a result, BiOI-MEPCM (1.5:1) demonstrated the roughest surface among the three BiOI-MEPCM samples.

### 3.2. Chemical Characterizations and Magnetic Response

**Figure 3:**
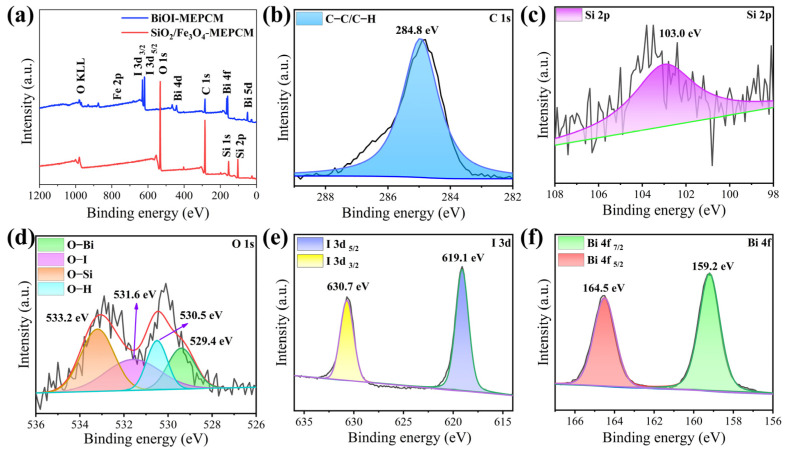
(**a**) Survey XPS spectra of phase-change microcapsules. (**b**–**f**) High-resolution XPS spectra of BiOI-MEPCM.

The chemical compositions of the BiOI-MEPCM were characterized by XPS, and the corresponding results are shown in Figure 3. The elements C, O, and Si were identified from the survey XPS spectrum of the SiO_2_/Fe_3_O_4_-MEPCM and are attributed to the SiO_2_ shell (Figure 3a). The BiOI-MEPCM spectrum shows not only the characteristic peaks of C, O, and Si elements but also I and Bi elements, which are derived from the deposited BiOI nanosheets. The high-resolution C 1s, with a binding energy of 284.8 eV, corresponded to the characterization background and were set as an internal reference to calibrate the high-resolution XPS spectra of the BiOI-MEPCM (Figure 3b). The deconvoluted, high-resolution Si 2p spectrum of BiOI-MEPCM exhibited one peak with a binding energy of 103.0 eV (Figure 3c). The high-resolution O 1s spectrum was deconvoluted into four peaks at 529.4, 530.5, 531.6, and 533.2 eV, which corresponded to O–Bi, O–H, O–I, and O–Si, respectively (Figure 3d). Moreover, the high-resolution I 3d and Bi 4f spectra showed two peaks with binding energies of 619.1 and 630.7 eV for I 3d and 159.2 and 164.5 eV for Bi 4f (Figure 3e,f). Fe was not detected in the survey XPS spectra due to a low content of Fe on the shell of the SiO_2_/Fe_3_O_4_-MEPCM and BiOI-MEPCM.

**Figure 4:**
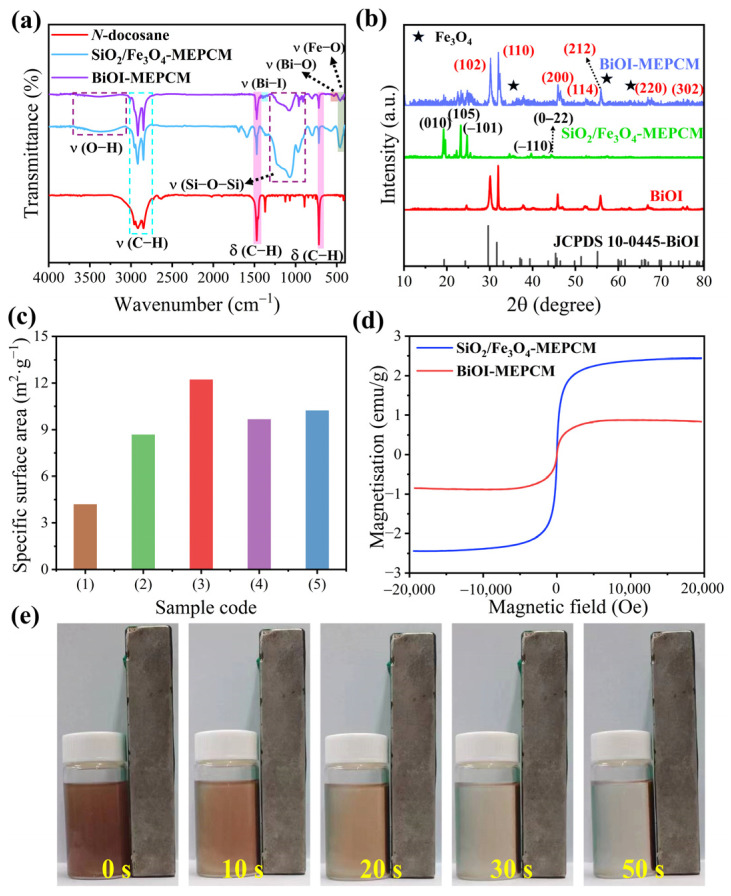
(**a**) FTIR spectra and (**b**) XRD patterns of *n*-docosane, pure BiOI, and phase-change microcapsules. (**c**) Specific surface area of (1) SiO_2_/Fe_3_O_4_-MEPCM, (2) pure BiOI, (3) BiOI-MEPCM (0.5:1), (4) BiOI-MEPCM (1:1), and BiOI-MEPCM (1.5:1). (**d**) Magnetic hysteresis loops of SiO_2_/Fe_3_O_4_-MEPCM and BiOI-MEPCM. (**e**) Digital photographs of BiOI-MEPCM dispersion under a magnetic field at different time.

FTIR spectroscopy was conducted to verify the chemical structure of the phase-change microcapsules. The FTIR spectra of pure *n*-docosane, SiO_2_/Fe_3_O_4_-MEPCM, and BiOI-MEPCM were recorded for comparison (Figure 4a). In the spectrum of pure *n*-docosane, the characteristic peaks near 728, 1476, 2850, and 2917 cm^−1^ are assigned to the characteristic vibration of C–H. For SiO_2_/Fe_3_O_4_-MEPCM and BiOI-MEPCM, the absorption bands corresponding to the *n*-docosane core were also observed from their FTIR spectra. The characteristic peaks attributed to the SiO_2_/Fe_3_O_4_ composite shell, including at 3430 cm^−1^ for O–H, 1071 cm^−1^ for Si–O–Si, and 574 and 527 cm^−1^ for Fe–O, were observed in the FTIR spectra of the phase-change microcapsules. Moreover, the characteristic peaks caused by the stretching vibrations of the Bi–O and Bi–I bonds appeared at 534 and 1375 cm^−1^ of the FTIR spectrum of BiOI-MEPCM, respectively, suggesting the presence of BiOI nanosheets in the microcapsules. Figure 4b shows the XRD patterns of pure BiOI and of the phase-change microcapsules. It can be seen that the pure BiOI presented diffraction peaks at 29.4°, 32.4°, 46.5°, 50.9°, 52.3°, 55.3°, and 76.1°, which are assigned to (102), (110), (200), (114), (212), (220), and (302), respectively, according to JCPDS card No. 10-0445 [20]. The SiO_2_/Fe_3_O_4_-MEPCM exhibited diffraction peaks at 19.2°, 23.2°, 24.7°, 39.7°, and 44.3°, attributed to the *n*-docosane core, and at 35.3°, 56.6°, and 62.3°, corresponding to the inverse spinel Fe_3_O_4_. For BiOI-MEPCM, the characteristic diffraction peaks of the *n*-docosane core, Fe_3_O_4_ nanoparticles, and BiOI nanosheets were all found in the XRD pattern without shifts, confirming that the two-step encapsulation process did not influence the crystalline structure of the core and shell materials. These results indicate the successful synthesis of BiOI-MEPCM with the desired micro-morphology and chemical compositions.

The specific surface area of the phase-change microcapsules was determined by an N_2_ adsorption–desorption experiment, and the corresponding results are presented in Figure 4c. The specific surface areas of the BiOI-MEPCM were higher than SiO_2_/Fe_3_O_4_-MEPCM. The nanostructural BiOI nanosheets contributed to such a large, specific surface area as they are capable of facilitating the adsorption and photocatalysis of the BiOI-MEPCM for water treatment. Nevertheless, the pure BiOI had a lower specific surface area than the BiOI-MEPCM due to the agglomeration of pure BiOI. Introducing Fe_3_O_4_ nanoparticles contributed a magnetic response to the phase-change microcapsules. As is shown in Figure 4d, a hysteretic behavior with low magnetic retentivity and coercivity was observed from the phase-change microcapsules in the magnetic field. This phenomenon suggests a superparamagnetic nature of the phase-change microcapsules. The magnetization saturation of the SiO_2_/Fe_3_O_4_-MEPCM and BiOI-MEPCM was determined to be 0.88 and 2.44 emu/g, respectively. The higher magnetization saturation of the SiO_2_/Fe_3_O_4_-MEPCM is due to the higher content of Fe_3_O_4_ nanoparticles in SiO_2_/Fe_3_O_4_-MEPCM compared to the BiOI-MEPCM. Nevertheless, the BiOI-MEPCM obtained a good magnetic response. The digital photograph in Figure 4e shows that the BiOI-MEPCM can be easily distributed in 30 s on the inside wall of a glass bottle by a magnet. This result indicates the feasible recyclability of BiOI-MEPCM using magnetism for water treatment and wastewater recovery.

### 3.3. Thermophysical Property

**Figure 5:**
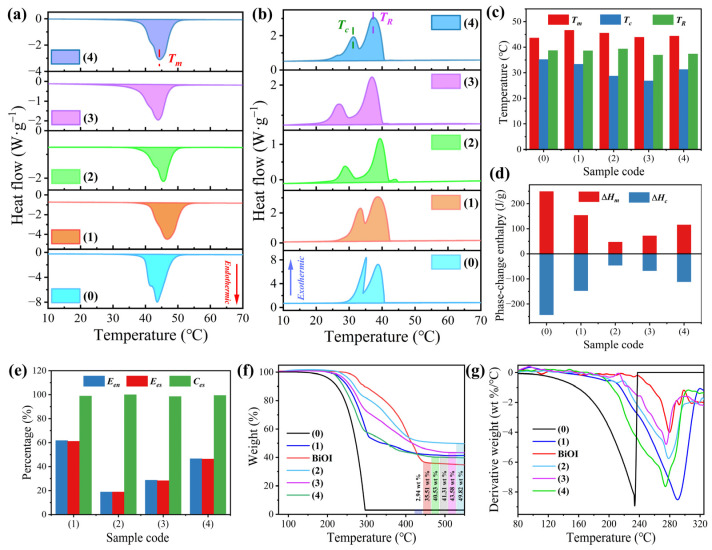
(**a**) Heating and (**b**) cooling DSC thermograms, (**c**) phase-change temperatures, (**d**) phase-change enthalpies, (**e**) encapsulation parameters, (**f**) TGA thermograms, and (**g**) DTG thermograms of (0) pure *n*-docosane, (1) SiO_2_/Fe_3_O_4_-MEPCM, (2) BiOI-MEPCM (1.5:1), (3) BiOI-MEPCM (1:1), (4) BiOI-MEPCM (0.5:1), and pure BiOI.

The thermophysical properties of pure *n*-docosane and the phase-change microcapsules were determined by DSC. The corresponding curves are presented in Figure 5a,b. The phase-change temperatures of pure *n*-docosane were determined at 43.63 °C for the melting temperature ™, 35.18 °C for the crystallization temperature (*T_c_*), and 38.74 °C for the rotator-phase-transition temperature (*T_R_*) (Figure 5c). The formation of the rotator phase of pure *n*-docosane is due to the weak intermolecular interaction that occurs during the phase transition of *n*-docosane from liquid to solid [35]. Compared to pure *n*-docosane, the SiO_2_/Fe_3_O_4_-MEPCM showed similar phase-change behaviors in the melting and crystallization processes, indicating that the phase transition processes of the encapsulated *n*-docosane were not influenced by the SiO_2_/Fe_3_O_4_ composite shell (Figure 5a,b). However, all the BiOI-MEPCM exhibited similar melting behaviors but showed little difference in crystallization behavior compared to the pure *n*-docosane. The decreased *T_c_* of the BiOI-MEPCM may be due to the geometric confinement of the micro-sized capsules, which confines the molecular movement of the *n*-docosane core and restricts its crystallization. As a result, the phase-change temperatures of the phase-change microcapsules slightly fluctuated compared to the pure *n*-docosane. As is shown in Figure 5d, pure *n*-docosane, as a typical, paraffin-type SLPCM, has a high latent heat capacity with a melting enthalpy (Δ*H_m_*) of 248.6 J/g and a crystallization enthalpy (Δ*H_c_*) of 243.3 J/g. However, the two-step encapsulation process caused a visible reduction in the phase-change enthalpies due to the encapsulation of the *n*-docosane core with the inert SiO_2_/Fe_3_O_4_ composite shell and BiOI nanosheets. The Δ*H_m_* and Δ*H_c_* of the SiO_2_/Fe_3_O_4_-MEPCM were determined to be 153.6 J/g and 147.2 J/g, respectively (Figure 5d). Following the surface deposition of the BiOI nanosheets, the BiOI-MEPCM showed a further reduction in phase-change enthalpies. The Δ*H_m_*s of BiOI-MEPCM (1.5:1), BiOI-MEPCM (1:1), and BiOI-MEPCM (0.5:1) were determined to be 46.8, 71.3 and 115.7 J/g, respectively.

The encapsulation parameters of the phase-change microcapsules, including the encapsulation ratio (*E_en_*, %), encapsulation efficiency (*E_es_*, %), and heat energy storage capability (*C_es_*, %), were determined by the following equations:

(1) $$E_{en}=\frac{\Delta H_{m,MEPCM}}{\Delta H_{m,SLPCM}}\times 100\%$$

(2) $$E_{es}=\frac{\Delta H_{m,MEPCM}+\Delta H_{c,MEPCM}}{\Delta H_{m,SLPCM}+\Delta H_{c,SLPCM}}\times 100\%$$

(3) $$C_{es}=\frac{(\Delta H_{m,MEPCM}+\Delta H_{c,MEPCM})\times \Delta H_{m,SLPCM}}{(\Delta H_{m,SLPCM}+\Delta H_{c,SLPCM})\times \Delta H_{m,MEPCM}}\times 100\%$$

where Δ*H_m,MEPCM_* and Δ*H_m,SLPCM_* are the melting enthalpies of the phase-change microcapsules and pure *n*-docosane, respectively, and Δ*H_c,MEPCM_* and Δ*H_c,SLPCM_* are the crystallization enthalpies of the phase-change microcapsules and pure *n*-docosane, respectively. Based on Equations (1) and (2), the *E_en_* and *E_es_* are highly related to the phase-change enthalpies of the phase-change microcapsules. As a result, SiO_2_/Fe_3_O_4_-MEPCM exhibited a maximum *E_en_* (61.78%) and *E_es_* (61.15%) among the phase-change microcapsule samples due to having the lowest shell material content of the microcapsule samples (Figure 5e). *C_es_* is a parameter for evaluating the reversible phase transition level of the encapsulated SLPCM in microcapsules [36]. The microcapsule size influences the heat energy storage capability. A large microcapsule size provides a broad inner space for the normal phase-change of the SLPCM. Otherwise, the phase transition of the encapsulated SLPCM does not occur due to the confinement effect on crystallization. As is shown in Figure 5e, although the deposition of nanosheets increased the shell content and then reduced the *E_en_* and *E_es_* of BiOI-MEPCM, all the BiOI-MEPCM samples obtained a high *C_es_* of more than 98%, suggesting that over 98% of the *n*-docosane core could proceed with a reversible, solid–liquid phase transition for storing and releasing heat energy.

A TGA was performed to determine the thermal stability of the phase-change microcapsules. The corresponding results are shown in Figure 5f,g. Pure *n*-docosane and SiO_2_/Fe_3_O_4_-MEPCM were found to exhibit a one-step thermal behavior because of the evaporation of *n*-docosane in the heating process (Figure 5f). However, a complicated thermal degradation process was observed from the BiOI-MEPCM samples due to the thermal decomposition of BiOI in addition to the evaporation of the *n*-docosane core. Compared to the pure *n*-docosane, all the phase-change microcapsules exhibited a higher content of char yield, which can be attributed to the SiO_2_, Fe_3_O_4_, and Bi_2_O_3_. In addition, there was a distinct improvement in the maximum degradation temperature for all phase-change microcapsules (Figure 5g). This result indicates the encapsulation of *n*-docosane with SiO_2_/Fe_3_O_4_ shell and BiOI nanosheets can provide a tight container to delay the evaporation of the *n*-docosane core.

### 3.4. Photocatalytic Activity

**Figure 6:**
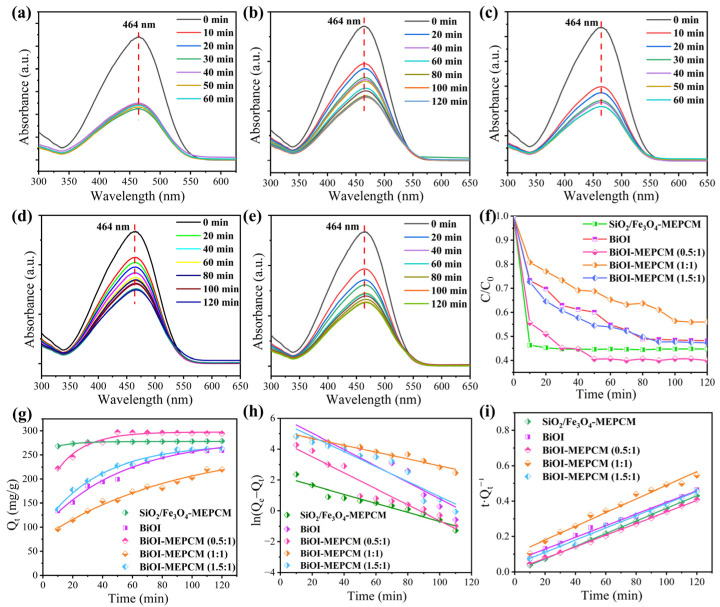
UV–vis absorption spectra of methyl orange during the adsorption process with (**a**) SiO_2_/Fe_3_O_4_-MEPCM, (**b**) BiOI, (**c**) BiOI-MEPCM (0.5:1), (**d**) BiOI-MEPCM (1:1), and (**e**) BiOI-MEPCM (1.5:1). (**f**) Adsorption ability of methyl orange during the adsorption process with BiOI and phase-change microcapsules. (**g**) *Q*_t_ of BiOI and phase-change microcapsules as a function of adsorption time for adsorbing methyl orange. Linear kinetic adsorption plots of methyl orange using (**h**) pseudo-first-order and (**i**) pseudo-second-order kinetic models for BiOI and phase-change microcapsules.

Methyl orange, Congo red, and tetracycline were selected as three representative organic contaminants to evaluate the photocatalytic activity of the phase-change microcapsules and the pure BiOI as a control for wastewater treatment. Before the evaluation of photocatalytic, the adsorption performance of the phase-change microcapsules was estimated in a dark environment. Figure 6a–e show the UV–vis absorption spectra of methyl orange solution during the adsorption process with the BiOI and phase-change microcapsules. It should be noted that the characteristic peak intensity corresponding to methyl orange decreased sharply with an increase in the adsorption time, indicating that methyl orange can be adsorbed onto the surface of the BiOI and phase-change microcapsules. The adsorption ability of the BiOI and phase-change microcapsules is attributed to the electrostatic interactions between the positively charged methyl orange and the negatively surface-charged BiOI and SiO_2_. On the other hand, the abundant hydroxyl groups on the SiO_2_/Fe_3_O_4_ composite shell can adsorb methyl orange through a hydrogen-bonding interaction. Therefore, all samples exhibited an adsorption ability for the removal of methyl orange. As is shown in Figure 6f, the BiOI-MEPCM (0.5:1) exhibited the best adsorption ability for methyl orange removal among all samples due to the synergism of the BiOI and SiO_2_. According to the SEM micrograph presented in Figure 2i, a low BiOI content led to an incomplete deposition of BiOI nanosheets onto the surface of the BiOI-MEPCM (0.5:1), resulting in a large surface area for the microcapsules to adsorb a larger amount of methyl orange. After adsorption for 120 min, all the methyl orange solutions maintained stability, indicating that the BiOI and phase-change microcapsules achieved an adsorption equilibrium. Therefore, a 120 min adsorption time was adopted before the evaluation of the photocatalytic activity.

Pseudo-first-order and pseudo-second-order kinetic models were used to evaluate the adsorption kinetic mechanism of the phase-change microcapsules by using the following equations [37]:

(4) $$\mathrm{Pseudo}-\mathrm{first}-\mathrm{order}:\;\ln(Q_{e}\;-\;Q_{t})=\ln Q_{e}\;-\;k_{1}\cdot t$$

(5) $$\mathrm{Pseudo}-\mathrm{second}-\mathrm{order}:\;\frac{t}{Q_{t}}=\frac{1}{k_{2}\cdot Q_{e}^{2}}+\frac{t}{Q_{e}}$$

where *Q*_e_ (mg/g) and *Q*_t_ (mg/g) represent the adsorption capacity at equilibrium and at adsorption time *t*, respectively, and *k*_1_ (min^−1^) and *k*_2_ (g·mg^−1^·min^−1^) refer to the equilibrium rate constant in the pseudo-first-order and pseudo-second-order kinetic models, respectively. *Q*_t_ can be determined by the following equation:

(6) $$Q_{t}=\frac{V(C_{0}-C)}{m}$$

where *V* represents the volume of the methyl orange solution, *C*_0_ and *C* refer to the concentrations of the methyl orange solution before initial absorption and after absorption time *t*, respectively, and *m* is the mass of the phase-change microcapsules. Figure 6g shows the adsorption capacity of the phase-change microcapsules as a function of adsorption time for adsorbing methyl orange. All microcapsule samples exhibited a rapid increase in adsorption capacity. Compared to the BiOI-MEPCM and pure BiOI samples, the SiO_2_/Fe_3_O_4_-MEPCM exhibited a faster adsorption time to achieve equilibrium adsorption within 20 min through a hydrogen-bonding interaction. Nevertheless, the BiOI-MEPCM (0.5:1) had the highest adsorption capacity (294.4 mg/g) among the microcapsule samples due to having the largest specific surface area (Figure 4c). Figure 6h,i display the kinetic plots achieved from the analysis with the pseudo-first-order and pseudo-second-order models. The corresponding kinetic parameters are summarized in Table 1. A regression coefficient of over 0.97 was obtained from the pseudo-second-order kinetic model in the adsorption process of methyl orange by different adsorption samples. This result is much close to one in comparison with the pseudo-first-order model, which suggests that the kinetic mechanism can be described by the pseudo-second-order model.

**Figure 7:**
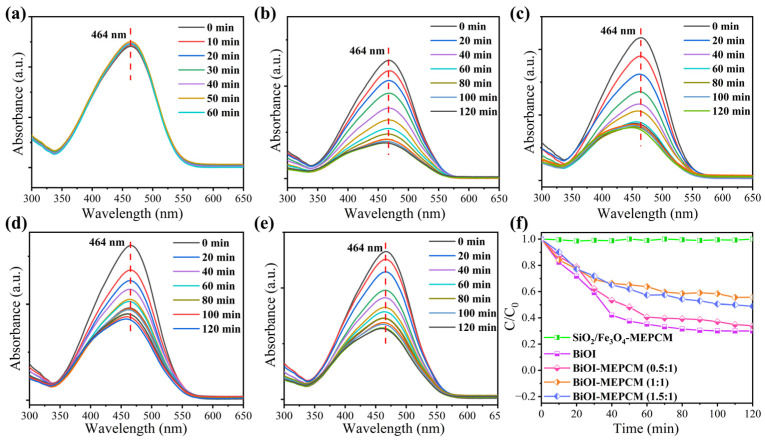
UV–vis absorption spectra of methyl orange during the photocatalytic process with (**a**) SiO_2_/Fe_3_O_4_-MEPCM, (**b**) BiOI, (**c**) BiOI-MEPCM (0.5:1), (**d**) BiOI-MEPCM (1:1), and (**e**) BiOI-MEPCM (1.5:1). (**f**) Photocatalytic degradation of methyl orange during the photocatalytic process with BiOI and phase-change microcapsules.

**Figure 8:**
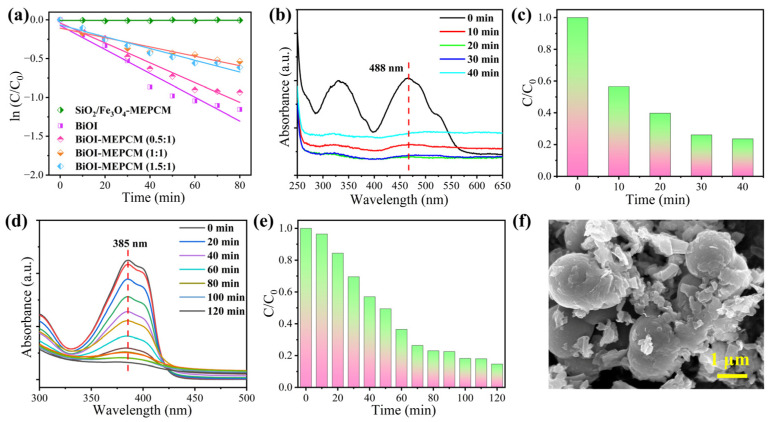
(**a**) ln (*C*/*C*_0_) as a function of irradiation time during the photocatalytic process. (**b**) UV–vis absorption spectra and (**c**) photocatalytic degradation of Congo red during the photocatalytic process with BiOI-MEPCM. (**d**) UV–vis absorption spectra and (**e**) photocatalytic degradation of tetracycline during the photocatalytic process with BiOI-MEPCM. (**f**) SEM micrograph of BiOI-MEPCM after stirring by a rotor-stator homogenizer.

Figure 7a–e depict the representative UV–vis absorption spectra of methyl orange solution during the photocatalytic process with the BiOI and phase-change microcapsules. The absorbance intensity of methyl orange remained unchanged during the photocatalytic process with the SiO_2_/Fe_3_O_4_-MEPCM (Figure 7a), suggesting no photocatalytic activity for the SiO_2_/Fe_3_O_4_ composite shell (Figure 7f). However, the absorbance intensity of methyl orange decreased gradually with an increase in illumination time during the photocatalytic process with the BiOI and phase-change microcapsules (Figure 7b–e). This phenomenon indicates an effective photocatalytic activity of the BiOI and BiOI-MEPCM. Generally, visible light irradiation can generate electrons and positive holes in the conduction and valence bands of BiOI, respectively [22,38]. The photogenerated electrons and positive holes transfer onto the surface of BiOI, facilitating the formation of superoxide radicals. These superoxide radicals act as oxidants to attack the methyl orange molecules, resulting in the photocatalytic degradation of methyl orange. As is shown in Figure 7f, BiOI, BiOI-MEPCM (0.5:1), BiOI-MEPCM (1:1), and BiOI-MEPCM (1.5:1) obtained degradation rates of 69.90%, 66.16%, 44.32%, and 51.12%, respectively, after continuous illumination for 120 min. It is understandable that the BiOI exhibited superior photocatalytic activity to BiOI-MEPCM due its higher content of pure BiOI, which can degrade more methyl orange molecules. Although BiOI-MEPCM (1:1) and BiOI-MEPCM (1.5:1) have a higher content of BiOI nanosheets in the microcapsules than BiOI-MEPCM (0.5:1), some BiOI nanosheets were self-aggregated (Figure 2d,f), decreasing the specific surface areas and active sites of the BiOI nanosheets for degrading methyl orange. As a result, the BiOI-MEPCM (0.5:1) achieved the best photocatalytic activity among the three BiOI-MEPCM samples. The pseudo-first-order model was adopted to evaluate the photocatalysis kinetic mechanism of the phase-change microcapsules using the following equation [39]:

(7) $$-\ln(C\;-\;C_{0})=kt$$

where *C*_0_ and *C* represent the initial absorption value of methyl orange before irradiation and absorption value after irradiation time *t*, respectively, and *k* refers to the corresponding degradation rate constant. According to the fitting curves shown in Figure 8a, the degradation rate constants, *k*, for SiO_2_/Fe_3_O_4_-MEPCM, pure BiOI, BiOI-MEPCM (0.5:1), BiOI-MEPCM (1:1), and BiOI-MEPCM (1.5:1) were determined to be 0, 0.0125, 0.0104, 0.0048, and 0.0065 min^−1^, respectively. This result confirms a non-photocatalytic degradation ability for the SiO_2_/Fe_3_O_4_-MEPCM. On the other hand, the *k* value of BiOI-MEPCM (0.5:1) was close to that of pure BiOI, demonstrating a good photocatalytic degradation ability of BiOI-MEPCM (0.5:1). Moreover, as a presentative sample, BiOI-MEPCM (1:1) exhibited good photocatalytic activity for degrading Congo red and tetracycline (Figure 8b–e). The characteristic peak intensity at 488 and 385 nm, which corresponded to Congo red and tetracycline, respectively, decreased sharply with the increase in illumination time (Figure 8b,d). Only 23.60% of Congo red remained after illumination for 40 min (Figure 8c). Regarding tetracycline, the BiOI-MEPCM obtained a degradation rate of 85.31% after illumination for 120 min (Figure 8e). These results confirm that the BiOI-MEPCM designed in this study exhibits great potential for the solar photocatalytic degradation of organic pollutants in wastewater. To confirm the mechanical stability of the BiOI-MEPCM for practical applications in water treatment, the BiOI-MEPCM (1:1) was stirred using a rotor-stator homogenizer (FJ200-SH, Shanghai Specimen Model Factory, China) for 30 min at a homogenization rate of 5000 rpm. Compared to the SEM micrograph in Figure 2, the BiOI-MEPCM exhibited a similar morphology without any crack after the homogenization test (Figure 8f), suggesting that the shell can offer an adequate mechanical strength of the BiOI-MEPCM for wastewater treatment.

### 3.5. Heat-Energy Storage Performance

**Figure 9:**
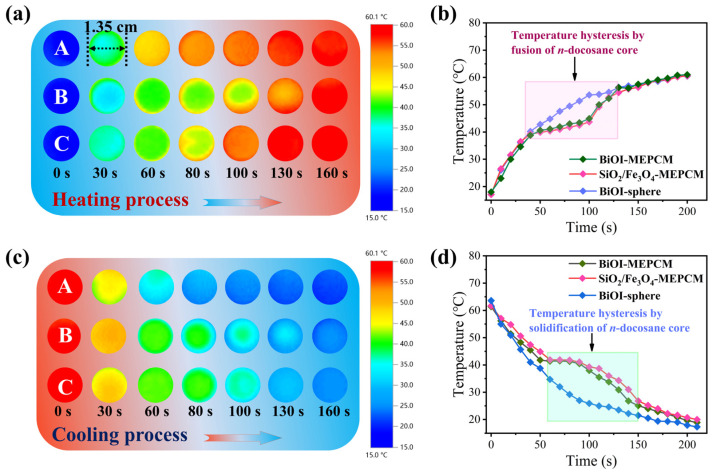
(**a**,**c**) Representative thermographic images and (**b**,**d**) temperature evolution of (A) BiOI-sphere, (B) SiO_2_/Fe_3_O_4_-MEPCM, and (C) BiOI-MEPCM during heating and cooling processes.

An infrared thermographic method was selected, using BiOI-MEPCM (1:1) as a representative sample to evaluate the heat energy storage performance of the BiOI-MEPCM for waste heat recovery. The SiO_2_/Fe_3_O_4_-MEPCM and BiOI-sphere were selected as two controls. Figure 9a,c show representative thermographic images during the isothermal heating and cooling processes. As a control without an *n*-docosane core, the BiOI-sphere exhibited a rapid color change during the heating and cooling processes because sensible heat determines its temperature. However, the SiO_2_/Fe_3_O_4_-MEPCM and BiOI-MEPCM displayed a slower color change, indicating a retarded thermal response with the increase in heating or cooling time. This retarded thermal response is due to the latent heat storage and release by the encapsulated *n*-docosane to melt and freeze during the heating and cooling processes, respectively. For this reason, the temperature evolution of the SiO_2_/Fe_3_O_4_-MEPCM and BiOI-MEPCM exhibited two discernible temperature hysteresis regions in the range of 38.8–64.6 °C during the heating process and 41.9–25.1 °C during the cooling process (Figure 9b,d). These two temperature hysteresis regions are in accord with the melting and crystallization of encapsulated *n*-docosane, as presented in Figure 5. It should be noted that the SiO_2_/Fe_3_O_4_-MEPCM had a more significant temperature hysteresis than the BiOI-MEPCM in view of the high content of *n*-docosane in SiO_2_/Fe_3_O_4_-MEPCM, which can store and release a larger amount of heat energy. These results confirm that the BiOI-MEPCM obtained a desired function for heat absorption and reutilization through the solid–liquid phase transition of the *n*-docosane core, indicating a potential application in absorbing waste heat from wastewater and releasing it as latent heat for reutilization.

### 3.6. Shape Stability and Thermal Reliability

**Figure 10:**
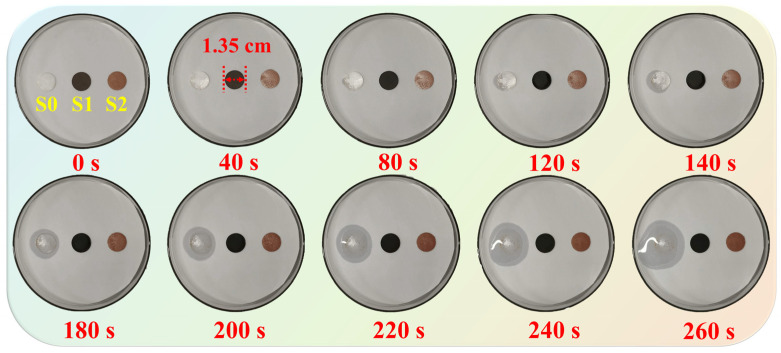
Digital photographs of (S0) pure *n*-docosane, (S1) SiO_2_/Fe_3_O_4_-MEPCM, and (S2) BiOI-MEPCM during the isothermal heating process.

The shape stability of the BiOI-MEPCM was evaluated by an isothermal experiment at 90 °C using BiOI-MEPCM (1:1) as a representative sample and pure *n*-docosane and the SiO_2_/Fe_3_O_4_-MEPCM as two controls. Digital photographs of the samples during the isothermal heating process are shown in Figure 10. Pure *n*-docosane melted as the heating time increased; therefore, the sample lost its shape, exhibiting a poor shape stability. Compared to the pure *n*-docosane, the SiO_2_/Fe_3_O_4_-MEPCM and BiOI-MEPCM demonstrated a stable shape during the heating process. No leakage or discernible exudation was found in the phase-change microcapsule samples. It is understandable that when *n*-docosane was encapsulated in the microcapsules, the SiO_2_/Fe_3_O_4_ composite shell and BiOI nanosheets maintained the liquid *n*-docosane core in the microcapsules for heat energy storage and prevented it from flowing away, further confirming that the phase-change microcapsules provide good mechanical stability under a pressure of 5.0 MPa.

**Figure 11:**
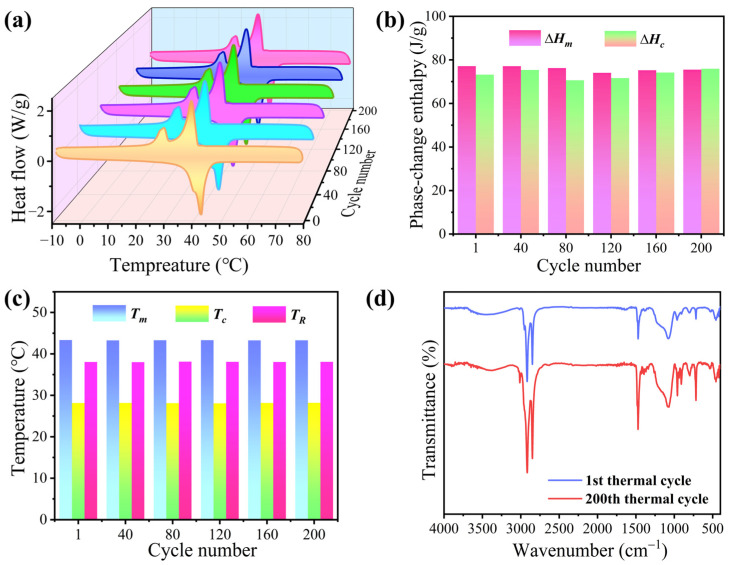
(**a**) DSC thermograms, (**b**) phase-change enthalpies, and (**c**) phase-change temperatures of BiOI-MEPCM (1:1) at every 40 heating-cooling thermal cycles. (**d**) FTIR spectra of BiOI-MEPCM (1:1) before and after thermal cycles.

The thermal reliability of the phase-change microcapsules was characterized by DSC. BiOI-MEPCM (1:1) was selected as a representative sample to verify their thermal reversibility and long-term stability for heat energy storage and release. Figure 11a shows the DSC curves of BiOI-MEPCM obtained from the microcapsules at every 40 heating–cooling thermal cycles between −10 and 70 °C. BiOI-MEPCM shows uniform DSC curves during the 200 heating–cooling thermal cycles. Only a slight fluctuation was observed from the phase-change enthalpies (Figure 11b) and temperatures (Figure 11c). The phase-change enthalpies fluctuated around ±4.3 J/g, and the phase-change temperatures varied within 0.14 °C during the thermal cycling experiment. Furthermore, the BiOI-MEPCM exhibited highly similar FTIR spectra before and after the thermal cycling experiment (Figure 11d). No visible difference in the location and intensity of the characteristic peaks was observed in the FTIR spectra for BiOI-MEPCM. These results indicate that the BiOI-MEPCM has an excellent thermal reliability for performing multicycle, solid–liquid phase transitions for the long-term storage and release of heat energy in waste heat recovery.

## 4. Conclusions

In summary, we designed and developed a novel type of multifunctional phase-change microcapsule, BiOI-MEPCM, to provide an effective solution for the recovery of waste and wastewater treatment. The phase-change microcapsules were synthesized using *n*-docosane as an SLPCM core and a SiO_2_/Fe_3_O_4_ composite as a base shell through in-situ interfacial polycondensation with the assistance of a Fe_3_O_4_ nanoparticle as a Pickering emulsion stabilizer, followed by the surface deposition of BiOI nanosheets. Benefiting from the *n*-docosane core, the BiOI-MEPCM achieved phase-change enthalpies of 46.8–115.7 J/g to absorb waste heat from wastewater through a solid–liquid phase transition. The deposited BiOI nanosheets led to photocatalysis, enabling the microcapsules to degrade organic contaminants in wastewater such as methyl orange, Congo red, and tetracycline. Owing to the magnetic response derived from Fe_3_O_4_ nanoparticles, the BiOI-MEPCM exhibited good separability and recyclability by magnetic separation. Moreover, BiOI-MEPCM demonstrated an outstanding phase-change reversibility, thermal cycling stability, and shape stability due to the tight SiO_2_/Fe_3_O_4_ composite shell and the BiOI nanosheets. This study provides a promising approach to designing and developing multifunctional phase-change microcapsules for waste heat recovery and wastewater treatment.

## Figures and Tables

**Table 1:** Kinetic parameters of pseudo-first-order and pseudo-second-order models for methyl orange adsorption of phase-change microcapsules.

Adsorption Sample	Pseudo-First-Order Model		Pseudo-Second-Order Model	
	*k*_1_ (min^−1^)	*R* ^2^	*k*_2_ (g·mg^−1^·min^−1^)	*R* ^2^
SiO_2_/Fe_3_O_4_-MEPCM	0.02929	0.9268	0.00787	1
BiOI	0.05324	0.8851	0.00024	0.9901
BiOI-MEPCM (0.5:1)	0.05190	0.9274	0.00107	0.9989
BiOI-MEPCM (1:1)	0.02218	0.9624	0.00021	0.9774
BiOI-MEPCM (1.5:1)	0.04858	0.8931	0.00035	0.9927

## Data Availability

The raw/processed data required to reproduce these findings cannot be shared at this time as the data form part of an ongoing study.
